# Isolating High Antimicrobial Ability Lignin From Bamboo Kraft Lignin by Organosolv Fractionation

**DOI:** 10.3389/fbioe.2021.683796

**Published:** 2021-05-26

**Authors:** Jinyan Yun, Liao Wei, Wei Li, Duqiang Gong, Hongyu Qin, Xiujing Feng, Guojiang Li, Zhe Ling, Peng Wang, Baishuang Yin

**Affiliations:** ^1^College of Animal Science and Technology, Jilin Agricultural Science and Technology University, Jilin, China; ^2^Children's Hospital of Nanjing Medical University, Nanjing, China; ^3^Co-innovation Center for Efficient Processing and Utilization of Forest Resources, College of Chemical Engineering, Nanjing Forestry University, Nanjing, China; ^4^State Key Laboratory of Pharmaceutical Biotechnology, Department of Sports Medicine and Adult Reconstructive Surgery, Nanjing Drum Tower Hospital, The Affiliated Hospital of Nanjing University Medical School, Nanjing, China

**Keywords:** lignin, bamboo, organosolv fractionation, antioxidation, antimicrobial property

## Abstract

Lignin from different biomasses possess biological antioxidation and antimicrobial activities, which depend on the number of functional groups and the molecular weight of lignin. In this work, organosolv fractionation was carried out to prepare the lignin fraction with a suitable structure to tailor excellent biological activities. Gel permeation chromatography (GPC) analysis showed that decreased molecular weight lignin fractions were obtained by sequentially organosolv fractionation with anhydrous acetone, 50% acetone and 37.5% hexanes. Nuclear magnetic resonance (NMR) results indicated that the lignin fractions with lower molecular weight had fewer substructures and a higher phenolic hydroxyl content, which was positively correlated with their antioxidation ability. Both of the original lignin and fractionated lignins possessed the ability to inhibit the growth of Gram-negative bacteria (*Escherichia coli* and *Salmonella*) and Gram-positive bacteria (*Streptococcus* and *Staphylococcus aureus*) by destroying the cell wall of bacteria *in vitro*, in which the lignin fraction with the lowest molecular weight and highest phenolic hydroxyl content (L3) showed the best performance. Besides, the L3 lignin showed the ability to ameliorate *Escherichia coli-induced* diarrhea damages of mice to improve the formation of intestinal contents *in vivo*. These results imply that a lignin fraction with a tailored structure from bamboo lignin can be used as a novel antimicrobial agent in the biomedical field.

## Introduction

Lignin is the most abundant naturally phenolic composition in the cell wall of biomass, which is covalently linked with carbohydrates (mainly hemicellulose) to form lignin-carbohydrate complexes (LCC) (Huang et al., 2018; Jiang et al., 2018; Dong et al., 2020a). LCC is regarded as the obstacle for the utilization of biomass to prepare bio-chemicals and bio-polymers, due to the fact that it degrades the polymers (cellulose and hemicellulose) into monosaccharides. Hence, various technologies have been carried out to remove or degrade lignin to break down the firm construction of biomass. During the treatment process, lignin is dissolved in solvent systems, which can be recovered and regenerated for further application to prepare lignin-based adhesives, films, carriers, fertilizer, et al. (Lateef et al., 2009; Sadeghifar and Ragauskas, 2020; Chen et al., 2021; Wang et al., 2021; Yu et al., 2021). In industry, lignin is mainly derived from black liquor produced in the kraft pulping process in papermaking, which is a by-product of pulp and paper making (Chen J. et al., 2020). Now, most of the black liquor is mainly used for energy supply and alkali recovery of papermaking, resulting in the waste of lignin resources. Therefore, the high-value utilization of kraft lignin (KL) from the pulping process has garnered attention in recent years (Dong et al., 2020b; Torres et al., 2020; Liu et al., 2021; Zhao et al., 2021).

In the cell wall of biomass, lignin is mainly formed by the units of syringyl, guaiacyl, and p-hydroxyphenyl, which are linked by β-O-4 aryl ethers, β-β (resinols), β-5 (phenylcoumarans), β-1 (spirodienones), and 5-5 and 4-O-5 linkages (Chen et al., 2018; Rencoret et al., 2019; Lin et al., 2020). During the cooking stage of pulp, these linkages suffer from degradation, resulting in lignin with a low molecule weight in solvent systems. Although the degradation of β-O-4 endows the obtained lignin with higher contents of phenolic and alcohol hydroxyl groups, the reactivity of lignin still fails to meet the requirement for further application (Tian et al., 2017; Liu et al., 2019). Hence, various technologies should be carried out to improve lignin's reactivity by improving the hydroxyl groups or reducing the molecular weight. Reported technologies mainly include thermal pyrolysis, reduction degradation, oxidation degradation, liquefied degradation, organosolv fractionation, et al. (Schutyser et al., 2018; Torres et al., 2020; Zheng Y. et al., 2021). Thermal pyrolysis has the ability to degrade the molecular weight of lignin, while the approach should be carried out at high temperatures (200–500°C). The reduction and oxidation degradation approach improves the functional groups in lignin, while the resulted lignin always contains different degraded products. Organosolv fractionation is a kind of solvent extraction approach, which is an excellent method to fractionate the lignin into the sample with the desired molecular weight and functional group content (Cui et al., 2014; Jiang et al., 2017; Allegretti et al., 2018).

Due to the existing phenolic and aliphatic hydroxyl groups, carboxyl groups, and methoxy groups in lignin, lignin possesses antibacterial and antioxidant abilities due to carbohydrates from the biomass (Figueiredo et al., 2018; Tao et al., 2021). Hence, lignin has been widely used as an additive in preparing rubber, plastic, foam, elastomer, and other composite materials to improve the strength of the material or endow the materials with the desired property (Bian et al., 2017; Nair et al., 2017; Chen S. L. et al., 2020). In recent years, the biological activities of lignin, such as anti-inflammatory, antioxidation, anticancer, and antimicrobial activities, have been gradually discovered and explored. Anti-oxidation is the main biological activity of lignin, as the hydroxyl groups in lignin endow it with the ability to scavenge the free radicals and reactive oxygen species (ROS) of cells under oxidative stress (Pei et al., 2020; Yu et al., 2020; Gu et al., 2021; Solihat et al., 2021; Zheng L. et al., 2021). The increased level of ROS in the cell can cause many human diseases by changing oxidative stress. For example, Zheng L. et al. (2021) found that lignin in LCC possessed the ability to scavenge the intracellular and endogenous ROS in osteoblast cells (MC3T3-E1) in an inflammatory environment and then promote osteoblast differentiation. In the work of Pei et al. (2020), they found that lignin and lignin derivatives from different biomasses showed good performance in scavenging free radicals and ROS *in vitro* and *in vivo*, showing an excellent capability to inhibit the aggregation of polyQ protein by reducing the existing ROS in neuronal cells. Generally, the scavenging ability of lignin for ROS is dependent on the quantity of the hydroxyl groups. Hence, seeking lignin fractions with a sufficient amount of hydroxyl groups from kraft lignin is necessary to improve its antioxidation ability. It is well-known that the inhibiting ability of lignin against microorganism growth is related to its antioxidation ability (Yang et al., 2018; Xu et al., 2019). In addition, bacterial diarrhea is a common infectious disease in animal husbandry due to different bacteria, which has brought great losses to economic development. Although antibiotics are effective in preventing the occurrence and development of bacterial infections, their adverse effects will ultimately endanger human health through the food chain (Founou et al., 2016). Therefore, seeking an effective and green extract from different biomasses to treat bacterial infection-induced diarrhea is necessary. Many works showed that lignin from biomass had strong antimicrobial activities for pathogenic bacteria. Most of the evaluations were just carried out at a cell level *in vitro*. Hence, investigating the antimicrobial ability of lignin for pathogenic bacteria *in vivo* is necessary to understand if it can be applied in living animals.

In this work, an industrially feasible organosolv fractionation with different solvents by sequential precipitation was carried out to fractionate kraft lignin into the sample with low molecular weight and high functional groups content, which aimed to improve its antimicrobial activities for bacteria. The chemical structures of obtained lignin were characterized by various technologies. The *in vitro* antioxidant activities of the obtained lignin were evaluated by scavenging capabilities for free radicals. The antimicrobial ability was evaluated by analyzing the inhibition growth for Gram-negative bacteria [*Escherichia coli* (*E. coli*) and *Salmonella*] and Gram-positive bacteria [*Streptococcus* and *Staphylococcus aureus* (*S. aureus*)] *in vitro*. Moreover, the diarrheal mice model induced by *E. coli* were fed with a lignin fraction with the best antioxidant activity to evaluate its antimicrobial ability *in vivo*.

## Materials and Methods

### Materials and Reagents

Five-year-old moso bamboo was acquired from the planting bases in Yibin, China. The moso bamboo was cut into 3–5 cm chips for kraft pulping. The Gram-negative bacteria of *Escherichia coli* (*E. coli*, ATCC-25922), Salmonella (ATCC-14028), and Gram-positive bacteria of *Streptococcus* (ATCC-21059) and *Staphylococcus aureus* (*S. aureus*, ATCC-25923) were purchased from China Microbial Culture Preservation Center. Thirty-six Balb/c mice (weight ~20 g), half male and half female, were purchased from the Jitai Animal Products Agency in Chaoyang District. All used chemicals were analytical grade without being purified for use.

### Preparing the Kraft Lignin and Fractionation of Kraft Lignin

Kraft pulping with Na_2_S and NaOH was carried out to obtained the black liquor from the bamboo at the conditions of 20% sulfidity, 25% effective alkali charge, and a solid-to-liquid ratio of 1:6 at 160°C for 60 min, as per the work of Huang et al. (2017). The dissolved kraft lignin in black liquor was obtained by acid-precipitation. The obtained kraft lignin was washed by distilled water to remove the residual chemicals and air-dried for further use.

**Scheme 1:**
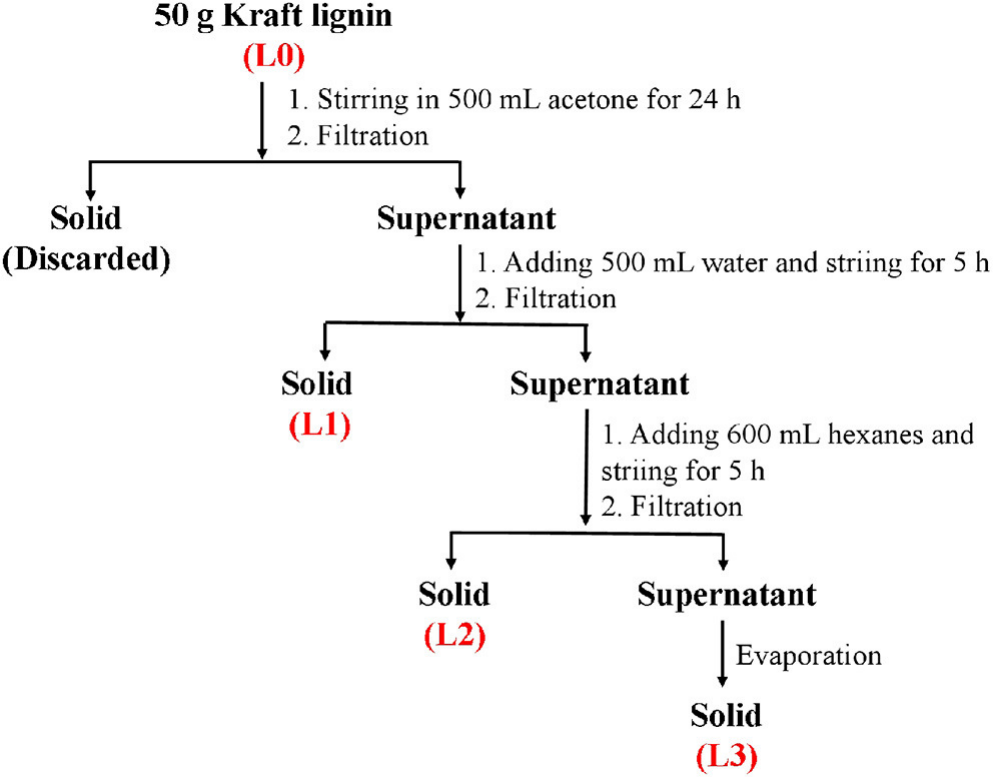
The process for fractionation of bamboo kraft lignin.

Fractionation of kraft lignin was carried out according to Scheme 1. Specifically, 50 g of air-dried kraft lignin was dispersed into 500 mL of acetone and stirred at 250 rpm for 24 h. After stirring, the solution was filtrated to obtain the supernatant. Then, 500 mL of distilled water was mixed with the obtained supernatant and stirred at 250 rpm for 5 h. The precipitate (L1) was obtained by filtration. The resulting supernatant was further mixed with 600 mL of hexanes and stirred at 250 rpm for 5 h. After stirring, the solution was filtrated to obtain the precipitate (L2) and supernatant. Then, rotary evaporation was carried out to remove the solvent in the supernatant to obtain the solid (L3). The obtained solids were dried in a vacuum drying oven and weighed to calculate their recovery yields.

### Characterization of Fractions From Kraft Lignin

The molecular weight of obtained lignin fractionations was analyzed by a Waters Gel Permeation Chromatography (GPC) instrument, which was equipped with a PL-gel 10 mm mixed-B 7.5 mm i.d. column and an ultraviolet detector. The GPC analysis was carried out using tetrahydrofuran as eluent at a 1 mL/min flow rate.

A Bruker AVANCE 600 MHz nuclear magnetic resonance (NMR) spectrometer equipped with a 5 mm BBO probe was used to characterize the structures of lignin fractionations. 2D-HSQC NMR analysis was carried out to get the information of linkages and units in lignins. The acquisition parameters were 160 transients from 1,024 data points (53 ms) for the F2 (^1^H) dimension and 256 data points (5.14 ms) for the F2 (^13^C) dimension. Quantitative ^31^P NMR analysis was carried out to determine the content of the hydroxyl groups and carboxyl groups in lignins. The detailed lignin preparation and acquisition parameters for NMR analysis were carried out according to the work of Pei et al. (2020).

### Antioxidant Activity Analysis of Fractions From Kraft Lignin

The *in vitro* antioxidant activity of the lignin fractionations were evaluated by scavenging capabilities on DPPH and ${\text{O}}_{2}^{\cdot -}$ radicals. For the DPPH scavenging assay, 3.0 mL of DPPH (0.2 mM) solution (dissolving in anhydrous ethanol) and 3.0 mL of ethanol were added to 3 mL of different concentrations of lignin solution (0.1–1 mg/mL, dissolving in anhydrous ethanol), respectively. After 30 min, the supernates of these mixtures were obtained to measure their absorbance at 517 nm, which were termed as D_sample_ and D_control_, respectively. In addition, 3.0 mL of ethanol was added to 3 mL of DPPH (0.2 mM) solution, and the absorbance of the mixtures was measured at 517 nm, termed as D_blank_. The DPPH radical scavenging rate (P) was calculated by the following equation:

$$\text{P}(\%)=\frac{\text{1}-({\text{D}}_{\text{sample}}{-\text{D}}_{\text{control}})}{{\text{D}}_{\text{blank}}}\times \text{100}\%$$

For the ${\text{O}}_{2}^{\cdot -}$ scavenging assay, 1.0 mL of lignin solution (0.1–1 mg/mL) was mixed with 4.5 mL of Tris-HCl solution (0.05 mol/L with pH 8.2) in a tube and incubated at 25°C for 20 min. Then, 0.5 mL of pyrogallol solution (45 mmol/L) was added to the tube. After the addition of pyrogallol solution, the absorbance of the mixture was measured at 325 nm every 30 s, termed as O_sample_. Meanwhile, 0.5 mL of water was used to replace the pyrogallol solution in the system. The supernate from this mixture was obtained to measure its absorbance at 325 nm, which was termed as O_control_. In addition, 1.0 mL of water ethanol was mixed with 4.5 mL of Tris-HCl solution (0.05 mol/L with pH 8.2) in a tube to measure the absorbance at 325 nm, termed as O_blank_. ${\text{O}}_{2}^{\cdot -}$ radical scavenging rate (R) was calculated by the following equation:

$$\text{R}(\%)=\frac{\text{1}-({\text{O}}_{\text{sample}}{-\text{O}}_{\text{control}})}{{\text{O}}_{\text{blank}}}\times \text{100}\%$$

### Antimicrobial Activity Analysis of Fractions From Kraft Lignin

#### Antimicrobial Activity Analysis by Microdilution Method

The antimicrobial activity of the lignin fractionations was tested against Gram-negative bacteria *E. coli* and *Salmonella* and Gram-positive bacteria of *Streptococcus* and *S. aureus* using the microdilution method. The lignin solution was mixed with the bacteria with a density of 1.0 × 10^6^ CFU/mL and incubated at 37°C for 12 h. The absorbance value of OD at 620 nm was measured by an enzyme plate analyzer, and the optimal inhibitory effect of lignin on bacteria was evaluated by calculating the reduction of the number of bacteria in the medium.

#### Antimicrobial Activity Analysis by Agar Media Method

The agar media method was also carried out to further analyze the antimicrobial activity of lignin. Specifically, the bacterial colonies formed on LB-agar plates calculated the inhibition rate of lignin for bacteria. A suspension of 200 colonies in LB agar medium was carried out in triplicate to analyze the antimicrobial activity of lignin with different concentrations (0.4–1.6 mg/mL) against each tested bacteria.

#### Extracellular Protein Assay

The lignin with the best antimicrobial activity was co-incubated with the tested bacteria for 12 h at a constant temperature of 200 rpm/min at 37°C. The bacterial suspension was centrifuged at 4,000 rpm/min for 10 min to get the supernatant and incubated bacteria. The absorbance value of the supernatant at 595 nm was determined with the BCA protein quantitative kit. Each sample was tested three times in parallel, and the bacterial liquid without extract was used as blank control to calculate the protein concentration of the supernatant. The incubated bacteria were dehydrated with graded ethanol and resuspended by ethyl alcohol for scanning electron microscope (SEM) analysis, which aimed to investigate the changes of the bacterial membrane.

### Animal Experiment

Thirty-six Balb/c mice (weight ~20 g), half male and half female, were used to analyze the *in vivo* antimicrobial activity of lignin. The animal experimental protocol was approved by the Animal Ethics Committee of Jilin Agricultural Science and Technology University (No: AWEC2017A01). In order to establish the mice diarrhea model, 200 μL of *E.coli* (1 × 10^9^ CFU/mL) was injected into the mice by intraperitoneal injection. The lignin solution was irrigated into the mice with a dosage of 2 mg/10 g body weight after diarrhea symptoms appeared in the mice. Seven days after the treatment, the diarrhea status, intestinal inflammation, and colon histological sections of the mice were observed to evaluate the antimicrobial activity of lignin *in vivo*.

### Statistical Analysis

All the results were expressed as mean ± standard deviation. Data were analyzed by one-way analysis of variance (ANOVA) using SPSS (2008) statistical software. Statistical analysis with different asterisks were expressed as ^*^p < 0.05, ^**^p < 0.01, ^***^p < 0.005, ^****^p < 0.001.

## Results and Discussion

### Recovery Yield and Molecular Weight of Lignin Fractions

In this work, the kraft lignin from bamboo was fractionated into three fractions (L1, L2, and L3) according to the described protocol in Scheme 1. The recovery yields of these fractions are shown in Table 1. It was shown that anhydrous acetone showed the highest ability to dissolve the lignin with a yield of 22.5%. The lignin fractions obtained from 50% acetone and 37.5% hexane fractionations had yields of 19.6 and 11.9%, respectively. The decreased yield of obtained lignin fractions might be due to the lower polarity of the solvent system, which can dissolve the low molecular weight lignin *via* aromatic π-π interactions (Cui et al., 2014; Jiang et al., 2017).

**Table 1:** The fractionation yields and molecular weight of lignin fractions.

	**Yield (%)^**a**^**	**Mw (g/mol)**	**Mn (g/mol)**	**PDI (Mw/Mn)**
L0	/	5,070	1,432	3.5
L1	22.5	7,260	3,090	2.3
L2	19.6	3,480	1,440	2.4
L3	11.9	1,810	950	1.9

a *Based on the initial L0 weight*.

The molecular weight of different lignin fractions was analyzed and is shown in Table 1. It was shown that organosolv fractionation with different solvents had the ability to fractionate the original lignin (L0) into the fractions with different molecular weights. The weight-average (Mw) and number-average (Mn) molecular weight of L0 were 5,070 and 1,432 g/mol, respectively. After sequent fractionation by anhydrous acetone, higher molecular weight lignin (L1) was obtained. While, with sequent fractionation by 50% acetone and 37.5% hexanes, lower molecular weight lignins were obtained with Mw values of 3,480 g/mol (L2) and 1,810 g/mol (L3), respectively. In addition, sequent fractionation dramatically reduced the heterogeneity of original lignin, which decreased the polydispersity index (PDI = Mw/Mn) of L0 from 3.5 to 2.3 and 1.9. This observation was also been found in the work of Cui et al. (2014), who isolated narrow fractions from softwood kraft lignin by fractional precipitation with hexanes and acetone. The higher monodispersity and lower molecular weight of L2 and L3 can endow these fractions with better reactivity and biological activity.

### NMR Characterization of Lignin Fractions

**Figure 1:**
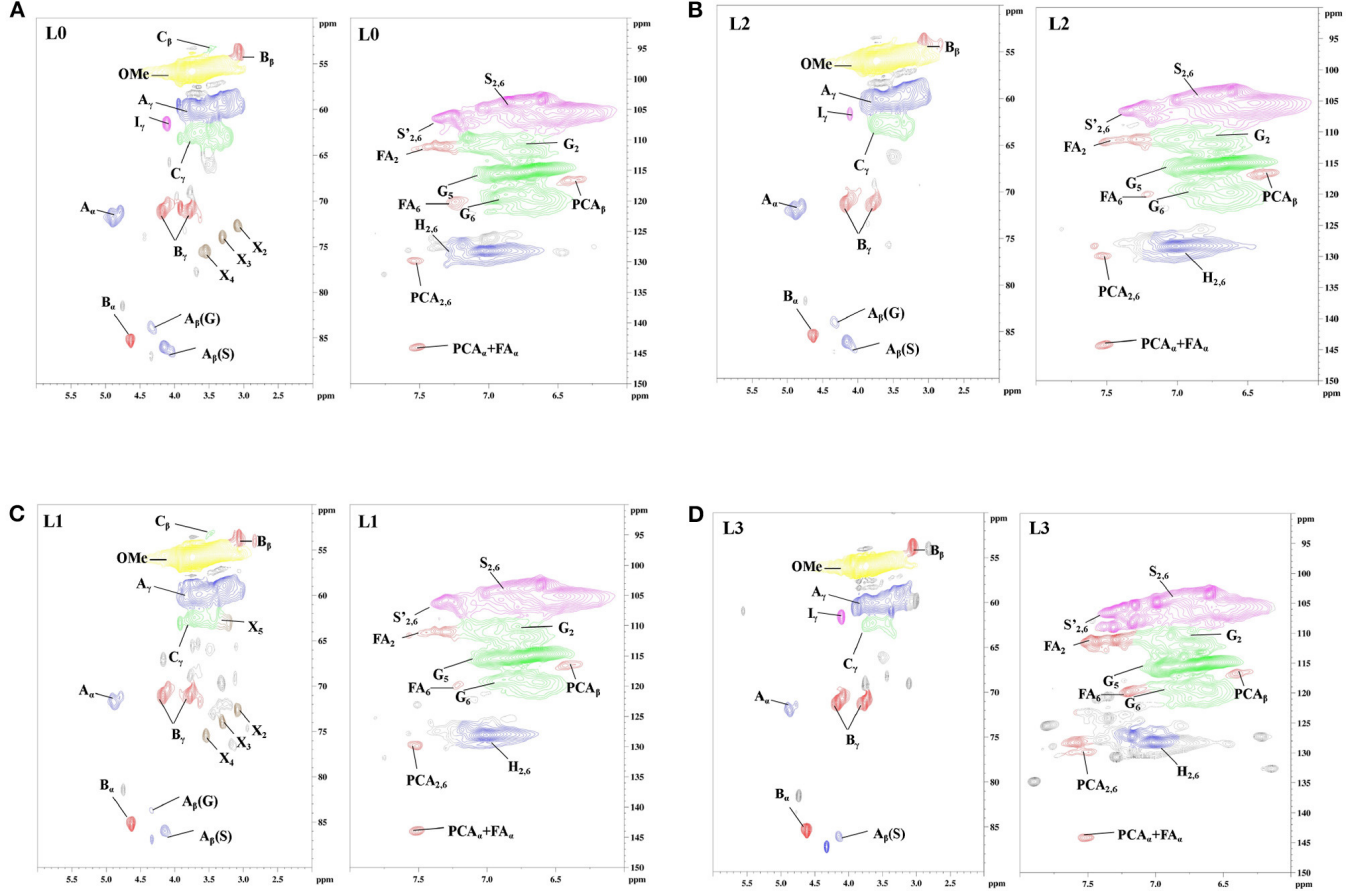
The 2D-HSQC NMR spectra of lignin (**A**: L0; **B**: L1; **C**: L2; **D**: L3).

**Figure 2:**
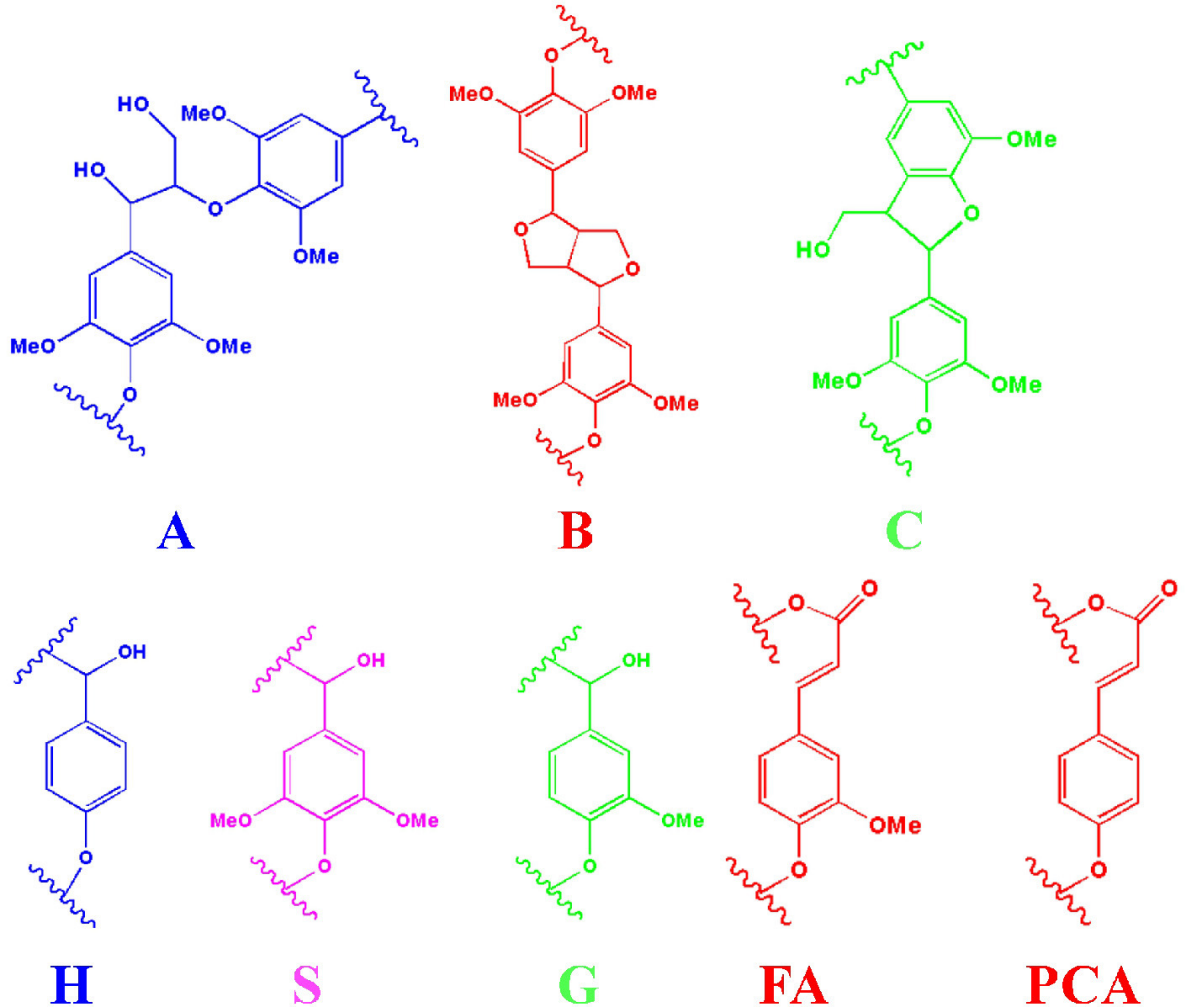
The main substructures of lignin in the spectra (A: *β*-O-4 substructures; B: resinol substructures; C: phenylcoumaran substructures; H: *p*-hydroxyphenyl units; S: syringyl units; G: guaiacyl units; FA: ferulate; PCA: *p*-coumarate).

In order to understand how the structural changes of lignin linkages of kraft lignin during organosolv fractionation with different solvents, 2D-HSQC NMR spectra of all lignin samples were obtained and are shown in Figure 1. The main lignin cross-signals (Supplementary Table 1) in the spectra are assigned by aligning the signals in the reported work of Wen et al. (2012), Dong et al. (2020b), and Pei et al. (2020). The main substructures of lignin in the spectra are depicted in Figure 2.

In the side-chain regions (δ_C_/δ_H_ 50–90/2.5–6.0) of all lignin's spectra, β-O-4 (A), β-β (B), and β-5 (C) substructures could be clearly observed by their corresponding C-H correlations. Specifically, the C_*a*_-H_*a*_ correlations for β-O-4, β-β, and β-5 were shown at δ_C_/δ_H_ 72.1/4.89, 84.9/4.65 and 86.9/5.51, respectively. Two C_β_-H_β_ signals of β-O-4 substructure were observed at δ_C_/δ_H_ 86.1/4.12 and 83.2/4.32, which were attributed to β-O-4 which was linked to the syringyl units (S) and guaiacyl units (G) in lignin. Signals at δ_C_/δ_H_ 71.4/4.20, 3.81 and 53.5/3.39 were attributed the C_β_-H_β_ correlations for β-β and β-5 substructures, respectively. In the side-chain regions (δ_C_/δ_H_ 160-90/8.0-6.0) of L3 spectra (Figure 1D), the signals for β-O-4 linked to G units were absent, indicating that this substructure might not have survived the organosolv fractionation process in the lignin fraction with the lowest molecular weight. In the aromatic region of all lignin spectra, the syringyl units (S), guaiacyl units (G), and *p*-hydroxyphenyl units (H) in fractionated lignins were also observed through their signals. S units, S units with C_a_=O groups (S′), and H units in lignin were identified by their C_2, 6_-H_2, 6_ signals at δ_C_/δ_H_ 104.2/6.75, 106.9/7.29, and 128.2/7.01, respectively. The G units showed C-H signals at δ_C_/δ_H_ 111.1/6.75, 116.4/6.73, and 119.9/6.83, which were attributed to the C_2_-H_2_, C_5_-H_5_, and C_6_-H_6_ correlations, respectively. In addition, the signals for the ferulate and *p*-coumarate, which are the phenolic compounds possessing antioxidant capacity (Jiang et al., 2018; Zheng L. et al., 2021), were also found in all lignin fractions by identifying their cross-peaks of correlations. Overall, the signals in all spectra indicated that the substructures of the obtained lignin fractions (L1, L2, and L3) did not change during the organosolv fractionation process.

The quantification of the substructures in lignin units were carried out to understand the changes of the interunit linkages of lignin. The amount of lignin substructures were calculated by their intensities in 2D-HSQC spectra and expressed per 100 Ar (Table 2). It was shown that the amount of β-O-4 substructures in F0, F1, F2, and F3 were 18.8/100 Ar, 13.5/100 Ar, 12.5/100 Ar, and 69.1/100 Ar, respectively. For the β-β and β-5 substructures in F1, F2, and F3, their amount also decreased as their molecular weight went down. These results indicated that sequent fractionation by anhydrous acetone, 50% acetone and 37.5% hexanes, was the approach to obtain lignin fractions with lower substructures along with their decreased molecular weight. In the work of Yang et al. (2018), they also found that the substructures content of different lignin fractions by organosolv fractionation exhibited decreasing trends with their molecular weight gradient.

**Table 2:** Quantitative amount of substructures in lignin fractions.

	**Amount of lignin substructures [100 Ar (C900)]**^**a**^			**Percentage of lignin unit (%)**^**b**^		
	**β-O-4 (A)**	**β-β (B)**	**β-5 (C)**	**S**	**G**	**H**
F0	18.8	5.6	0.9	48.8	25.4	25.9
F1	13.5	4.3	0.6	47.4	24.7	27.9
F2	12.5	2.1	0.6	50.8	24.7	23.5
F3	9.1	2.1	0.5	51.0	24.5	24.5

a *The amounts were calculated based the following formula: IC900 = 0.5 × IS_2, 6_ + IG_2_ + 0.5 × IH_2, 6_ IX% = IX/IC900 × 100%; where Ix is the integral value of a position signal in the lignin sub-linkages (β-O-4, β-β, and β-5) in the 2D spectra region*.

b *Molar percentages S + G + H = 100%*.

The functional groups of each lignin fractions were quantificationally analyzed from quantitative ^31^P NMR analyses and are shown in Table 3. For all the lignin fractions from L0, a decreased order for the contents of aliphatic hydroxyl and increased order for the contents of phenolic hydroxyl were consistent in the sequence of the fractionation process. Specifically, the aliphatic hydroxyl content of F1 was 3.21 mmol/g, which was higher than that of F2 (3.19 mmol/g) and F3 (2.04 mmol/g). The phenolic hydroxyl content of F1, F2, and F3 were in the increased order of 2.78, 2.89, and 3.66 mmol/g. These results were in agreement with the work of Cui et al. (2014) and Brodin et al. (2009), who reported that the amount of functional groups in the lignin fractions obtained by membrane filtration and organosolv fractionation were directly related to their molecular weight. In the work of Yang et al. (2018), the antioxidant and antimicrobial capacities of lignin were found to be related to the phenolic hydroxyl group in the lignin structure. Hence, it was speculated that the obtained lignin fraction of L3 with the highest amount of phenolic hydroxyl content might have the best biological activity.

**Table 3:** The contents of functional groups in lignin fractions (mmol/g).

	**Aliphatic hydroxyl**	**Phenolic hydroxyl**		**Total phenolic hydroxyl**	**COOH**
		**Condensed phenolic OH**	**Non-condensed phenolic OH**		
F0	2.98	1.12	1.59	3.71	0.12
F1	3.21	1.22	1.56	2.78	0.11
F2	3.19	1.23	1.66	2.89	0.12
F3	2.04	1.45	2.21	3.66	0.11

### Antioxidant Activity Analysis of Lignin Fractions

**Figure 3:**
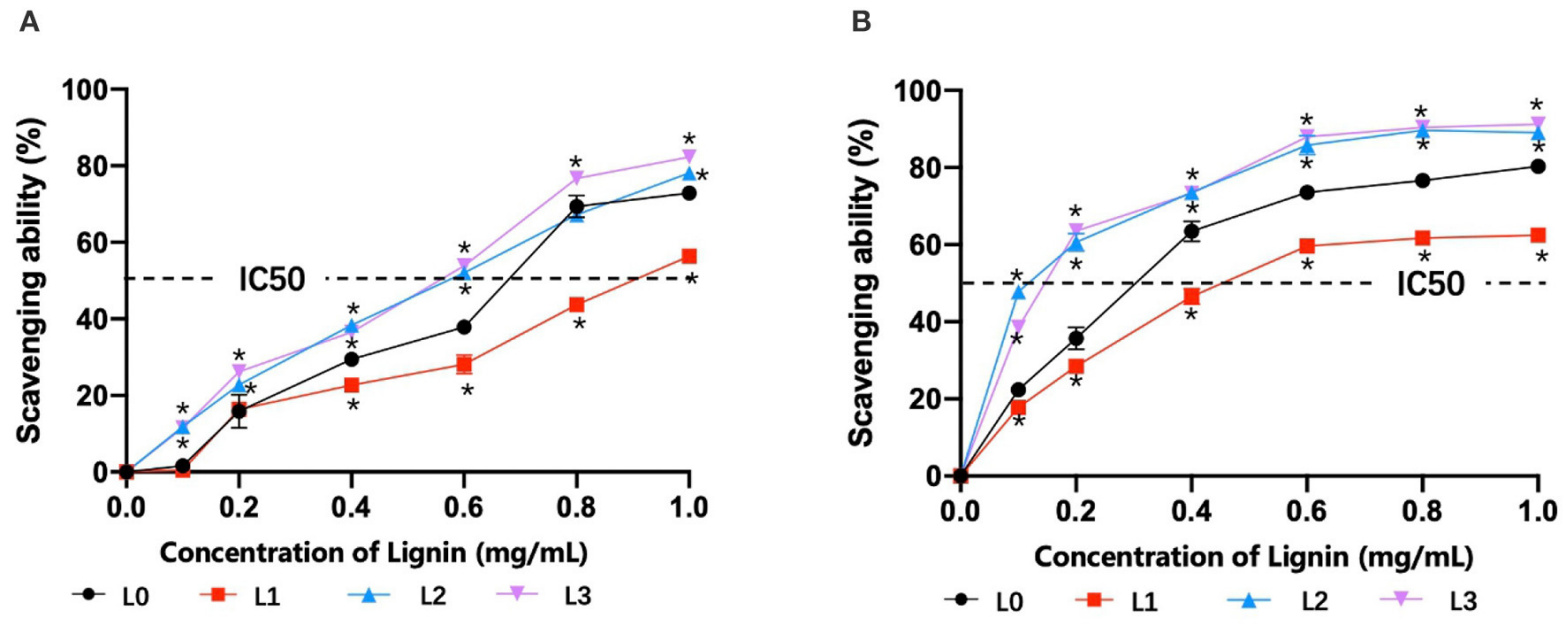
The scavenging abilities of lignin for DPPH radicals **(A)** and ${\text{O}}_{2}^{\cdot -}$ radicals **(B)** (*p < 0.05).

To analysis the antioxidant activity of lignin fractions, their scavenging abilities for DPPH and ${\text{O}}_{2}^{\cdot -}$ radicals were tested and are shown in Figure 3. It showed that the scavenging abilities for DPPH radicals (Figure 3A) and ${\text{O}}_{2}^{\cdot -}$ radicals (Figure 3B) were increased with the dosage of lignin fractions, in which L3 showed the best performances at higher concentrations. IC50 values, the concentration required for 50% scavenging of the free radical, are generally considered to evaluate the radical scavenging activity of the lignin. From the plotted line in Figures 3A,B, it was found that L2 and L3 possessed comparable values for DPPH radicals (0.09 and 0.11 mg/mL) and ${\text{O}}_{2}^{\cdot -}$ radicals (0.56 and 0.57 mg/mL), respectively, which was lower than that of L0 (0.29 mg/mL for DPPH radicals and 0.68 mg/mL for ${\text{O}}_{2}^{\cdot -}$ radicals) and L1 (0.46 mg/mL for DPPH radicals and 0.90 mg/mL for ${\text{O}}_{2}^{\cdot -}$ radicals). These results indicated that L2 and L3 had the best antioxidant activity, which was against with our initial speculation that L3 with the highest phenolic hydroxyl content would possess the best antioxidant ability. The reason for this phenomenon might be explained by the fact that the existing phenolic substances (PCA and FA) in lignin fractions also contribute to its antioxidant ability, in which their amount in L2 and L3 are different (Jiang et al., 2020; Zheng L. et al., 2021).

### *In vitro* Antimicrobial Behavior of Lignin Fractions

**Figure 4:**
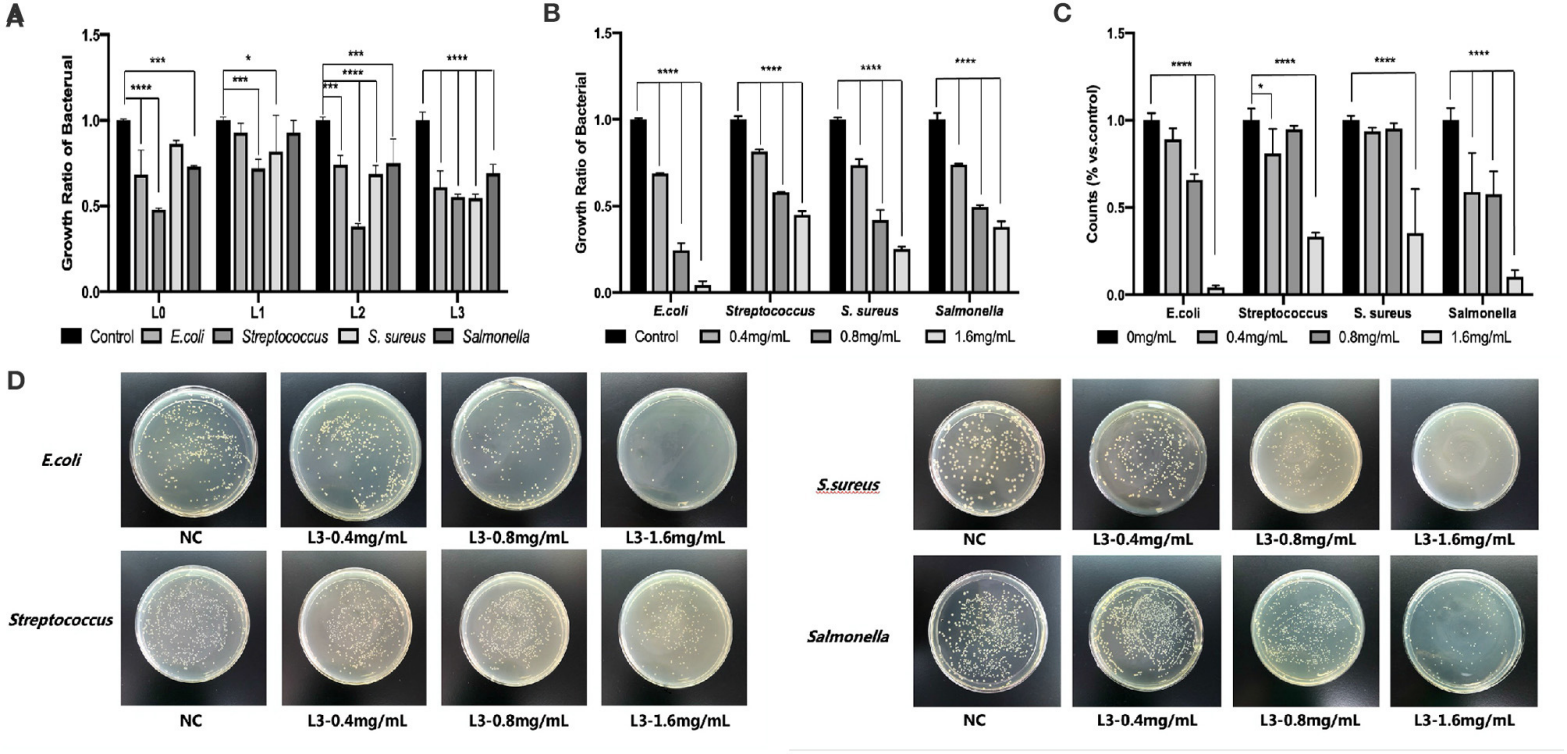
The antimicrobial behavior of lignin fractions by the microdilution method **(A)**, effect of different lignin concentrations of L3 on antimicrobial ability by the microdilution method **(B)** and by the agar media method **(C)**, effect of different lignin concentrations of L3 on the colony of different bacteria **(D)** (*p < 0.05; ***p < 0.001; and ****p < 0.0001).

Bacterial diarrhea is a common infectious disease from Gram-negative bacteria and Gram-positive bacteria in animal husbandry, which has brought great losses to economic development. Although antibiotics are effective in preventing the occurrence and development of bacterial infections, their adverse effects will ultimately endanger human health through the food chain (Founou et al., 2016). In order to demonstrate the antimicrobial activity of lignin fractions, *in vitro* assays by the microdilution method were performed using *E.coli, Salmonella, Streptococcus*, and *S. aureus*, which are common bacteria with strong virulence for diarrhea. Figure 4A shows that both the original lignin (L0) and fractionated lignins (L1, L2, and L3) displayed excellent inhibitory effects on all bacteria by significantly inhibiting their growth, of which L3 had the strongest antimicrobial activity. This might be due to its high content of phenolic hydroxyl groups and lowest molecular weight (Li et al., 2007). Therefore, the antimicrobial properties of lignin L3 were explored in detail in subsequent research.

Figure 4B shows that the L3 lignin significantly decreased the growth and reproduction of *E. coli, Salmonella, Streptococcus*, and *S. aureus*, which the degree of antimicrobial properties was dependent on the lignin's concentration by the microdilution method. In addition, the L3 lignin showed the best antimicrobial ability for *E. coli*, and a comparable ability for *Salmonella, Streptococcus*, and *S. aureus*. For example, an inhibition ratio of 95.61% was found for *E. coli* treated with 1.6 mg/mL of L3, which was higher than that for *Salmonella* (89.60%)*, Streptococcus* (66.62%), and *S. aureus* (64.68%). The remarkable inhibitory effect of the L3 lignin on different bacteria in a dose-dependent manner could also be observed from the statistical results (Figure 4C) from antimicrobial assays by the agar media method, which were calculated from colony-counting results in Figure 4D. Compared to the control group, a debasement of formed bacterial colonies was observed in the groups treated with the L3 lignin with increased concentrations from 0.4 mg/mL to 1.6 mg/mL, indicating the excellent antimicrobial property of the L3 lignin. For example, the colonies of *E. coli, Streptococcus, S. aureus*, and *Salmonella* were, respectively, decreased from 89, 81, 94, and 59% to 4, 33, 35, and 10% when they were incubated with the L3 lignin with concentrations from 0.4 to 1.6 mg/mL.

### Effect of the L3 Lignin on Bacterial Cell Wall

In the work of Medina et al. (2016) and Kaur et al. (2017), they reported that lignin had an inhibitory effect on *Bacillus subtilis, E. coli, Salmonella, Listeria, S. aureus*, and *Klebsiella*, which may be due to its greater influence on deconstructing the cell wall of bacteria, thus hindering the growth and reproduction of bacteria. In addition, the protein content in the supernatant reflected the damage degree of the bacterial cell wall (Chung and Chen, 2008; Rojas et al., 2018). To understand how L3 affects the cell wall of bacteria, the changes of bacterial cytoderm were investigated by analyzing the released protein content in culture solution.

**Figure 5:**
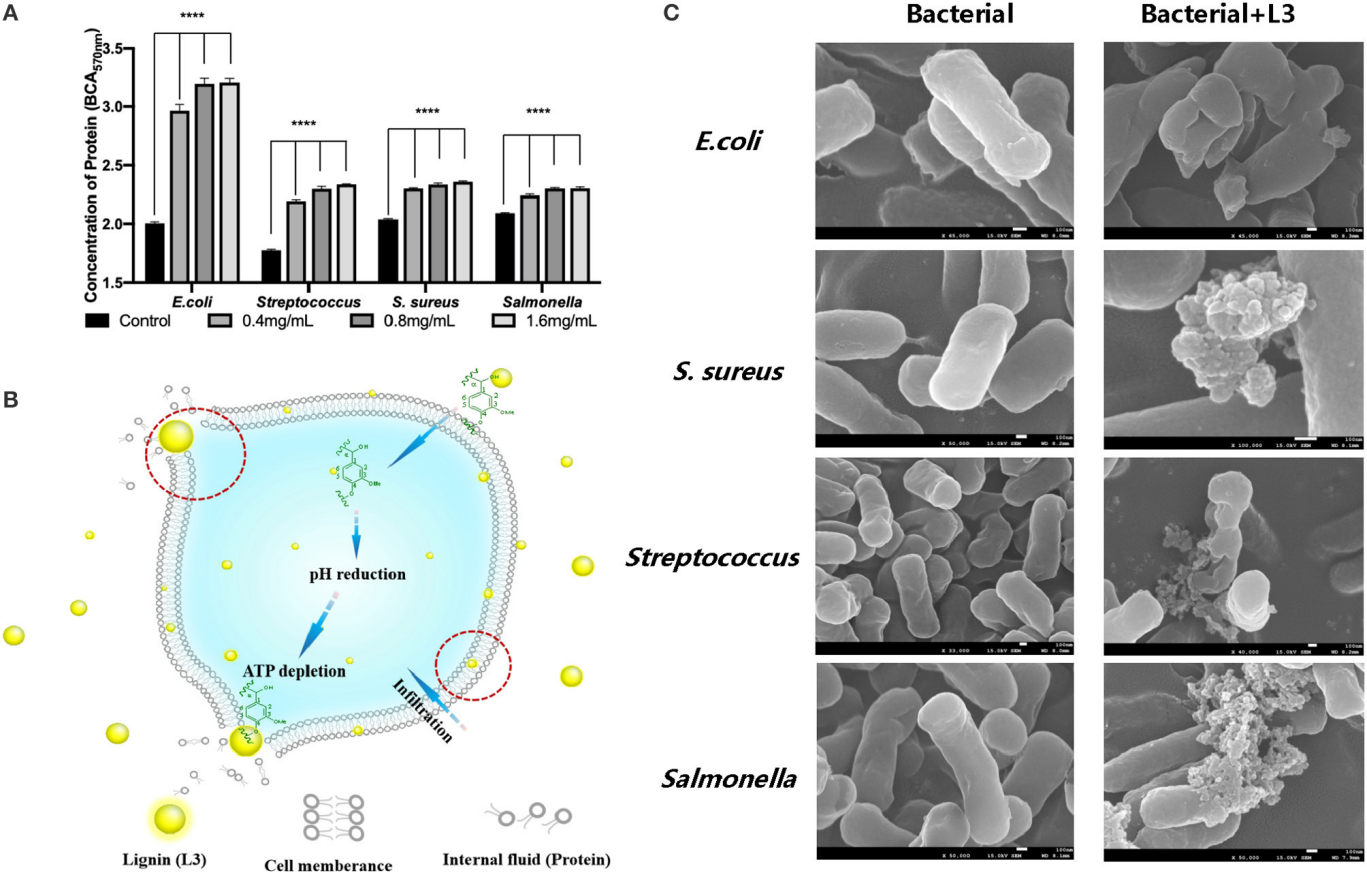
The protein content in the culture solution of bacteria treated by the L3 lignin **(A)**; the morphological image of the bacterial cell wall before and after treatment with the L3 lignin **(B)**; the proposed antimicrobial mechanism of L3 **(C)** (****p < 0.0001).

Figure 5A shows that the protein content of the blank group was maintained at a very low level without significant changes. After treatment with the L3 lignin, the protein content in the culture solution was significantly higher than that in the blank control group, indicating that lignin L3 caused damage to the cytoderm of the bacteria, and the degree of damage was proportional to the increased concentration. Under the action of lignin L3, the protein content in the supernatant increased in a dose-dependent manner, which indicated that the effect of pathogenic damage was strong, especially for *E. coli*. The damages of the different bacteria cell walls were also identified by their morphological changes (Figure 5B). The intact and smooth cell wall could be observed from the morphology images of normal bacterial cells. While, obvious damage resulting in the wizened membranes was formed for the bacteria after being treated with the L3 lignin. The antimicrobial effect of the L3 lignin may be due to the creation of a low pH environment on the cell membrane, which destroyed the proton dynamics of the cell membrane. Polyphenols in lignin have the capacity to damage cell walls by lysis, resulting in effective leakage of the internal fluid, which may be related to the strong antioxidant effects of lignin (Jiang et al., 2020). On the other hand, a large number of reactive oxygen species (ROS) are concentrated on the surface of lignin. When lignin makes contact with bacteria, ROS may release and induce oxidative stress by changing its normal redox physiological process (Lobo et al., 2010; Yang et al., 2018; Wang et al., 2019; Zheng L. et al., 2021). In this work, with the increase of lignin concentration, the content of soluble protein in the culture medium increased, indicating that the cell membrane of the tested bacteria was damaged and its normal life activities were affected. Hence, the antimicrobial mechanism of L3 was proposed and is shown in Figure 5C.

### *In vivo* Antimicrobial Behavior of the L3 Lignin

**Figure 6:**
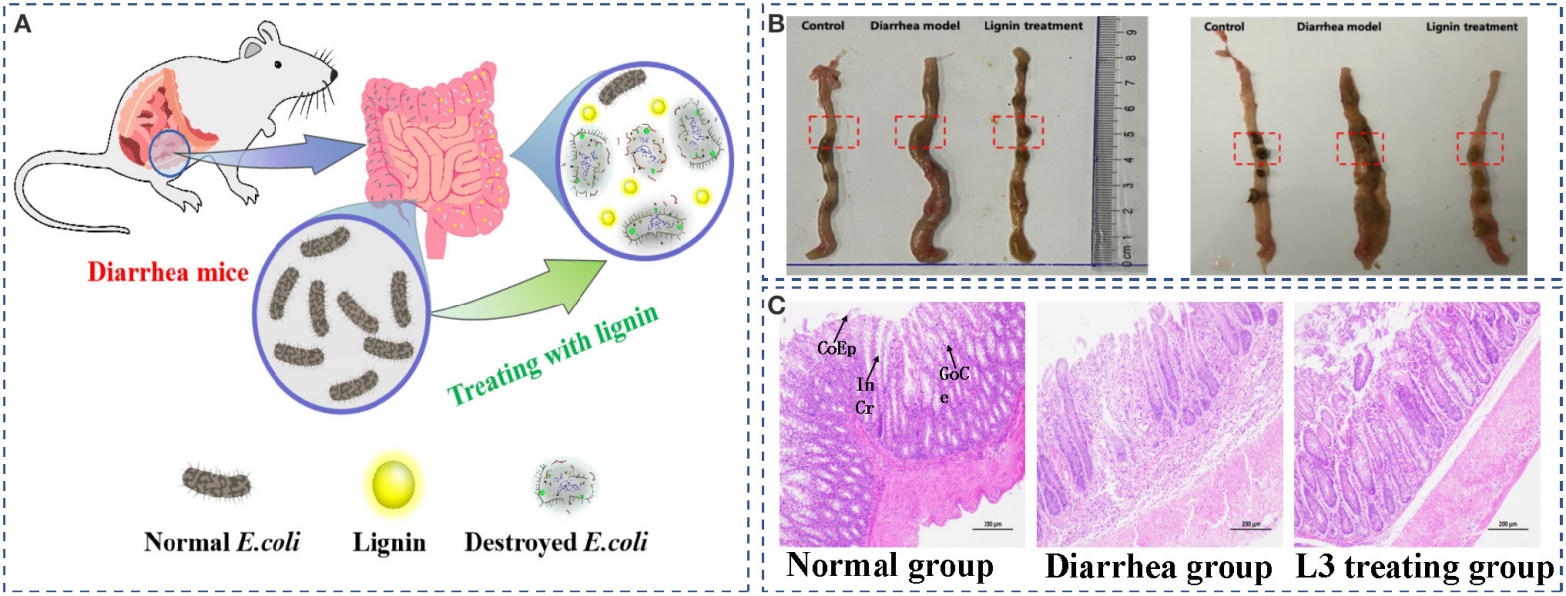
*E. coli*-induced diarrhea mice infection model treated by feeding with the L3 lignin **(A)**; the images of intestinal contents **(B)** and histopathological colon **(C)** of diarrhea mice before and after treatment with L3 lignin. Scale bar = 200 μm. CoEp, columnar epithelium; InCr, intestinal crypt; GoCe, goblet cell.

To evaluate the *in vivo* antimicrobial behavior of the L3 lignin, an *E. coli*-induced diarrhea mice infection model treated by feeding with the L3 lignin was performed (Figure 6A). The results of the necropsy (Figure 6B) showed that there was colon congestion and swelling in the model group, while the intestinal swelling and hyperemia of the mice treated with lignin were remitted, and inflammation was alleviated. Compared with the control group, the intestinal contents of mice in the diarrhea model group included yellow loose stool and were unformed. However, the diarrhea status of mice in the lignin treatment group was somewhat reduced, indicating that lignin could relieve the *E. coli*-induced diarrhea. Histopathological analyses (Figure 6C) were further carried out to investigate the effects of the L3 lignin on *E.coli*-induced diarrhea. The results of the histological features including crypt shortening, edema, mucosal erosions, and infiltration of inflammatory cells in lamina propria were observed in the diarrhea mice group (Wang et al., 2019). Unexpectedly, mice with diarrhea treated with the L3 lignin reflected reduced histological changes for pathological inflammation compared with the diarrhea mice group. The results indicated that the L3 lignin exerted obvious protective effects on bacterial infection-induced intestinal damage in mice. These results showed that the L3 lignin had the ability to ameliorate *E. coli* diarrhea damages, which showed an excellent *in vivo* antimicrobial behavior.

## Summary

In this work, the lignin fraction with decreased molecular weight and increased phenolic hydroxyl contents could be successfully prepared from bamboo kraft lignin by sequential organosolv fractionation with anhydrous acetone, 50% acetone and 37.5% hexanes. The antioxidation and antimicrobial properties of the lignin fractions were related to their inherent structural properties. Due to the excellent antioxidation and antimicrobial abilities *in vitro*, the L3 lignin fraction was able to ameliorate *E. coli-induced* diarrhea damages of mice to improve the formation of intestinal contents *in vivo*. All the obtained results indicated that the proposed organosolv fractionation was an excellent way to prepare the high antimicrobial ability lignin from bamboo kraft lignin, which can be further applied in different biomedical fields.

## Data Availability Statement

The original contributions presented in the study are included in the article/Supplementary Material, further inquiries can be directed to the corresponding authors.

## Ethics Statement

The animal experimental protocol was approved by the Animal Ethics Committee of Jilin Agricultural Science and Technology University (No: AWEC2017A01). Written informed consent was obtained from the owners for the participation of their animals in this study.

## Author Contributions

JY and LW undertook the experiments of antimicrobial activity and wrote the manuscript. WLi, DG, HQ, and XF completed the antimicrobial assay of the L3 lignin *in vivo*. ZL did the structural analysis of lignin fractions. GL, PW, and BY proposed the idea and revised the manuscript. All authors contributed to the article and approved the submitted version.

## Conflict of Interest

The authors declare that the research was conducted in the absence of any commercial or financial relationships that could be construed as a potential conflict of interest.
