# Supplementary material for: Isolating High Antimicrobial Ability Lignin From Bamboo Kraft Lignin by Organosolv Fractionation
Source: Front Bioeng Biotechnol. 2021 May 26;9:683796. doi: 10.3389/fbioe.2021.683796 (PMC8188334; doi:10.3389/fbioe.2021.683796)
Supplement: Supplementary file 1 [file Table_1.doc]

Table S1. Assignment of main cross-signals in the 2D-HSQC NMR spectra of lignin fractions

| Labels | δ_C_/δ_H_ | Assignment |
| --- | --- | --- |
| Lignin structure | | |
| C*_β_* | 53.1/3.49 | C*_β_*-H*_β_* in phenylcoumaran substructures (C) |
| B*_β_* | 53.5/3.39 | C*_β_*-H*_β_* in resinol substructures (B) |
| -OCH_3_ (OMe) | 55.9/3.73 | C-H in methoxyls |
| A*_γ_* | 59.6-60.8/3.37-3.72 | C*_γ_*-H*_γ_* in *β*-O-4 substructures(A) |
| I*_γ_* | 61.4/4.09 | C*_γ_*-H*_γ_* in cinnamyl alcohol end-groups (I) |
| B*_γ_* | 71.3/4.18,3.82 | C*_γ_*-H*_γ_* in resinol substructures (B) |
| A*_a_* | 72.1/4.89 | C*_a_*-H*_a_* in*β*-O-4 substructures(A) |
| A*_β_*_（G）_ | 83.2/4.32 | C*_β_*-H*_β_* in *β*-O-4 substructures linked to a G unit (A) |
| B*_a_* | 84.9/4.65 | C*_a_*-H*_a_* in resinol substructures (B) |
| A*_β_*_（S）_ | 86.1/4.12 | C*_β_*-H*_β_* in *β*-O-4 substructures linked to a S unit (A) |
| C*_a_* | 86.9/5.51 | C*_a_*-H*_a_* in phenylcoumaran substructures (C) |
| S_2,6_ | 104.2/6.75 | C_2,6_-H_2,6_ in etherified syringyl units (S) |
| S'_2,6_ | 106.9/7.29 | C_2,6_-H_2,6_ in syringyl units with C*_a_*=O groups (S') |
| G_2_ | 111.1/6.75 | C_2_-H_2_ in guaiacyl units (G) |
| FA_2_ | 111.1/7.34 | C_2_-H_2_ in ferulate (FA) |
| PCA*_β_* | 113.8/6.29 | C_8_-H_8_ in *p*-coumarate (PCA) |
| PCA_3,5_ | 116.2/6.77 | C_3_-H_3_ and C_5_-H_5_ in *p*-coumarate (PCA) |
| G_6_ | 119.9/6.83 | C_6_-H_6_ in guaiacyl units (G) |
| FA_6_ | 123.1/7.19 | C_6_-H_6_ in ferulate (FA) |
| H_2,6_ | 128.2/7.01 | C_2,6_-H_2,6_ in *p*-hydroxyphenyl units (H) |
| I*_β_* | 128.5/6.25 | C*_β_*-H*_β_* in cinnamyl alcohol end-groups (I) |
| I*_a_* | 128.5/6.47 | C*_a_*-H*_a_* in cinnamyl alcohol end-groups (I) |
| PCA_2,6_ | 130.1/7.48 | C_2,6_-H_2,6_ in *p*-hydroxyphenyl units (H) |
| PCA*_a_*, FA*_a_* | 144.7/7.46 | C*_a_*-H*_a_* in *p*-coumarate (PCA) and ferulate (FA) |
